# High-precision correlative fluorescence and electron cryo microscopy using two independent alignment markers^☆^

**DOI:** 10.1016/j.ultramic.2013.10.011

**Published:** 2014-08

**Authors:** Pascale Schellenberger, Rainer Kaufmann, C. Alistair Siebert, Christoph Hagen, Harald Wodrich, Kay Grünewald

**Affiliations:** a Oxford Particle Imaging Centre, Division of Structural Biology, Wellcome Trust Centre for Human Genetics, University of Oxford, Roosevelt Drive, Oxford OX3 7BN, UK; b Department of Biochemistry, University of Oxford, South Parks Road, Oxford OX1 3QU, UK; c Microbiologie Fondamentale et Pathogénicité, MFP CNRS UMR 5234, University of Bordeaux SEGALEN, 146 rue Leo Seignat, 33076 Bordeaux, France

**Keywords:** Fluorescent cryo microscopy, Electron cryo microscopy, Cellular tomography, Fluorescent microspheres, Correlative microscopy, Ad5-488, Adenovirus serotype 5 labelled with Alexa-488, CLEM, correlative light and electron microscopy, cryoEM, electron cryo microscopy, cryoET, electron cryo tomography, cryoFM, fluorescence cryo microscopy, EM, electron microscopy, FM, fluorescence microscopy, FWHM, full-width at half-maximum, HMM, high magnification montages, LM, light microscopy, LMM, low magnification montages, LN_2_, liquid nitrogen, LWD, long working distance, NA, numerical aperture, PSF, point spread function, ROI, region of interest, STED, stimulated emission depletion microscopy, σ^A^, accuracy of alignment, σ^C^, accuracy of fluorescent fiducial based coordinates transformation, σ^E^, accuracy of locating event of interest, σ^T^, total accuracy of correlation

## Abstract

Correlative light and electron microscopy (CLEM) is an emerging technique which combines functional information provided by fluorescence microscopy (FM) with the high-resolution structural information of electron microscopy (EM). So far, correlative cryo microscopy of frozen-hydrated samples has not reached better than micrometre range accuracy. Here, a method is presented that enables the correlation between fluorescently tagged proteins and electron cryo tomography (cryoET) data with nanometre range precision. Specifically, thin areas of vitrified whole cells are examined by correlative fluorescence cryo microscopy (cryoFM) and cryoET. Novel aspects of the presented cryoCLEM workflow not only include the implementation of two independent electron dense fluorescent markers to improve the precision of the alignment, but also the ability of obtaining an estimate of the correlation accuracy for each individual object of interest. The correlative workflow from plunge-freezing to cryoET is detailed step-by-step for the example of locating fluorescence-labelled adenovirus particles trafficking inside a cell.

## Highlights

• Vitrified mammalian cell were imaged by fluorescence and electron cryo microscopy.
• TetraSpeck fluorescence markers were added to correct shifts between cryo fluorescence channels.
• FluoSpheres fiducials were used as reference points to assign new coordinates to cryoEM images.
• Adenovirus particles were localised with an average correlation precision of 63 nm.

## 1 Introduction

Electron cryo microscopy (cryoEM) is a powerful technique to visualise molecules in their native state. The method consists of imaging biological samples in the frozen-hydrated state at cryogenic temperatures [1–3]. Vitrification, i.e. rapid freezing of specimens within an amorphous, non-crystalline, glass-like ice layer, allows the preservation of structures down to the molecular level in the native, hydrated state. This is of special importance for cellular samples that are sensitive to chemical fixation and dehydration [4,5]. CryoEM has been used to study a vast range of specimens, from isolated macromolecules to the complexity of a cell. Despite these tremendous capabilities, particular for cellular cryoEM, one major limitation remains: locating the subcellular event of interest with nanometre-scale precision on the three-millimetre-diameter, frozen-hydrated, EM grid.

A very promising approach to achieve this goal is to use position information from fluorescence microscopy. This method employs, for example, fluorescently labelled proteins, to assist with their subsequent location and identification in EM. This methodology is typically referred to as correlative light and electron microscopy (CLEM). Initially introduced for specimens at ambient or cultivation temperature (for recent reviews, see [6,7]), more recently it has been applied for frozen-hydrated specimens [8–10]. CLEM relies on a two-step approach. First, the regions of interest (ROI) are imaged and located with light and fluorescence microscopy techniques. Secondly, the samples are imaged in EM, and the ROIs are assigned based on the fluorescence data. Most proteins can be tagged with a fluorophore, and analysed specifically with a wide range of light microscopy techniques such as live-cell imaging, confocal microscopy, super-resolution, e.g.*,* stimulated emission depletion microscopy (STED) [11], or localisation microscopy [12–14]. The samples are either imaged by fluorescence microscopy before fixation (correlation with live-cell imaging), or after fixation. Expectedly, only post-fixation imaging allows for the confident correlation of fast, dynamic processes with EM, such as cell mobility, cytoskeleton dynamics, virus infection, and endocytosis. This limitation may be reduced by the use of rapid cryo immobilisation preparation procedures such as high-pressure freezing [15]. Most CLEM procedures use a two-step imaging workflow with a transfer step between microscopes dedicated for a particular modality (light microscopy (LM) or EM), as presented in this study. However, dedicated combined instruments which avoid the need for sample transfer exist [16,17]. CLEM was initially introduced for the analysis of sections of plastic embedded material, such as resin embedded samples or Tokuyashu type cryo sections in methyl cellulose. The technique was more recently applied to frozen-hydrated specimens, such as plunge-frozen purified complexes, and for guiding cryoET on whole vitrified cells [9,16,18–21].

Electron microscopists have made considerable efforts to facilitate the correlation across several orders of magnitude. For instance, special EM grids marked with numbers or letters recognisable in low magnification greatly assist the first steps of correlation. In most studies performed on plastic embedded samples, the fluorescence information only guides the correlation at a rather low-resolution scale, such as certain parts of a cell, and the re-location of the ROIs in EM remains somewhat approximate. Invasive immunolabelling or post-fixation contrasting methods are often used in order to better assign position information for the labelled molecules [22,23]. Only recently, a method using fluorescent electron-dense microspheres as CLEM fiducials achieved a correlation in the range of 100 nm, solely guided by fluorescence [24,25]. In these studies the bleed through from the FluoSpheres signal into the channel of the fluorescent protein of interest had been used to correct for shifts between the channels. With the approach used, the authors were able to follow the location of endocytic vesicles in resin embedded sections of yeast cells and to describe the endocytic process across several stages [26]. This impressive demonstration of the use of fluorescent fiducials for resin embedded samples poses the question of its applicability for cryo specimens.

Indeed, cryoLM has the great advantage to provide the location of the feature of interest at the exact same position on the EM grid without the need to compensate for sample deformation caused by the shrinkage and warping of plastic sections due to electron beam irradiation. At the same time, cryoLM/FM represents a greater challenge than conventional LM/FM imaging for a number of reasons. Firstly, the sample needs to be imaged whilst being kept below the de-vitrification point at about −135 °C [27]. With the development of dedicated LM cryo sample stages it became possible to perform such experiments [8,10,18–20]. Secondly, with the exception of in-column fluorescence microscopy implementations [16], the sample needs to be cryo-transferred to the electron microscope for subsequent EM imaging. This poses two risks, (i) the risk of damaging or losing the specimen, and (ii) the risk of ice contamination. Thus, in comparison to the substantial body of literature reporting the application of correlative studies on resin embedded samples, only a few studies have reported correlative cryoLM/EM. Moreover, correlation accuracy significantly below the micrometre precision has yet to be achieved [28].

Here, we describe a solution to improve the precision of correlative cryoLM/EM of plunge-frozen specimens. We present a workflow implementing (i) TetraSpeck fluorescent microspheres as markers for multi-channel cryo fluorescent image correction (as proposed in [24]), and (ii) fluorescent electron dense microspheres as reference points for an accurate correlation and transfer of coordinates between LM and EM. Besides giving an overview of the workflow, a detailed description of the practical steps is provided. The power of this approach is illustrated with a biological example; the high-precision location of adenovirus particles within infected cells.

## 2 Materials and methods

### 2.1 Purification and labelling of virus particles

First generation E1/E3-deleted Adenovirus serotype 5 viral vectors (Ad5) were produced by infecting twenty 15 cm dishes of HEK 293 cells (ATCC CRL-1573™) at a ratio of 100 physical particles per cell. Cells were continued to grow for 36 h until detachment was apparent. Cells were collected, pelleted and virus was extracted by freeze-thaw cycles and purified using double CsCl2-banding. Purified Ad5 particles were labelled using the Alexa-488 microscale protein labeling kit (Invitrogen) using the manufacturers protocol as described in detail in Martinez et al. [29] (Ad5-488).

### 2.2 EM grid preparation, infection of cells grown on EM grids

Gold finder EM grids with regular patterned holey carbon films (Protochips CF-2/1-2F1-Au) were glow-discharged for 20 s and coated by submersion in 100% poly-l-lysine solution (Sigma-Aldrich) for 30 to 60 min. Subsequently, the grids were coated with fluorescent microspheres that were used as fine correlation markers, *i.e* carboxylate-modified blue fluorescent FluoSpheres (350Ex/440Em) of 0.1 μm diameter, (Invitrogen). The application of a 1/4000 diluted FluoSphere solution directly onto the carbon support of the EM grid followed after 5 min with a washing step with PBS yielded the required density of ~5–15 FluoSpheres per high-magnification EM image. Subsequently, human bone osteosarcoma epithelial cells (U2OS) were grown on the grids in Dulbecco's modified medium (Invitrogen), supplemented with 10% (w/v) fetal calf serum (Sigma-Aldrich) for 15 to 24 h before infection. The growth medium was replaced after 10 to 15 h to remove unattached cells and cell debris. Fluorescently labelled adenovirus particles were adsorbed to human osteosarcoma cells (U2OS) at 37 °C for various amounts of time. Prior to vitrification 1 μl of 15 nm colloidal gold particles (Aurion) and 2 μl of a 1/20 diluted solution of TetraSpeck microspheres (0.2 μm diameter, showing four well-separated excitation/emission peaks, 360/430 nm (blue), 505/515 nm (green), 560/580 nm (orange) and 660/680 nm (dark red), Invitrogen) were added directly onto the grid. The density of TetraSpeck microspheres must be low enough to allow the detection of the green fluorescence of adenovirus particles (Ad5-488) in the FM, but high enough that sufficient fluorescent markers (~5–15) are found in a single grid square. Excess of buffer was removed by manual blotting from the non-carbon side of the grid, and cells were vitrified by plunge-freezing in a liquid nitrogen-cooled ethane/propane mixture [30]. Samples were stored submersed in liquid nitrogen for later analysis.

### 2.3 Fluorescence cryo microscopy

For fluorescent microscopy, an AxioObserver Z1 inverted microscope (Zeiss) equipped with 20× (NA 0.4) and 63× (NA 0.75, working distance 1.7 mm) air objectives, a motorised stage (Märzhäuser), and a monochrome AxioCam MRm camera (Zeiss) was used. A mercury arc lamp (HXP) was used for fluorescent excitation with the following band pass filter cubes (AHF): 482/18 (Ex), 520/28 (Em) (green); 560/40 (Ex) and 630/75 (Em) (red); 387/11 (Ex), 447/60 (Em) (blue). Zeiss AxioVision 4.3 software was used to control the microscope stage, filter cubes, shutters and camera. Fluorescent and phase contrast imaging at cryogenic temperatures was performed as described previously [19]. Briefly, a cryostage^2^ cryo sample stage (MPI Biochemistry, Martinsried [18]) was mounted onto the light microscope's motorised stage. This setup employs a liquid nitrogen (LN_2_) automated pumping system (Norhof LN_2_ microdosing system series 900). Samples were transferred from liquid nitrogen storage only when the temperature of the microscope cryo sample stage was below −170 °C. Image acquisition with the 63× objective lenses was performed in the sequence: red, green, blue and each channel was focused separately.

The point spread function (PSF) of images taken with the 63× objective at cryogenic temperature was calculated by measuring the green fluorescence of Ad5-488 labelled virus particles (90 nm diameter) and the red fluorescence of FluoSpheres (0.2 μm diameter, Invitrogen). The resulting resolution measured as full-width at half-maximum (FWHM) of the PSF was about 450 nm and 560 nm for the green and red channels, respectively. Images acquired with the 63× objective resulted in an effective pixel size of 103 nm. This samples the PSF well enough allowing the positions of sufficiently small point-like objects to be localised with sub-pixel accuracy, which is crucial for the subsequent correlation steps described below.

### 2.4 Electron cryo microscopy and tomography data collection

#### 2.4.1 Electron cryo microscopy

Electron cryo microscopy was performed at 300 keV, using a F30 ‘Polara’ electron microscope (FEI). Low-dose, zero-loss images were acquired with SerialEM [31] in EFTEM mode with a Gatan 964 Quantum energy filter operated with a 20 eV wide energy selecting slit. Images were recorded at nominal magnifications of 5600×, 9300× and 41,000× sampling the specimens at calibrated pixel sizes of 3.65 nm, 2.10 nm and 0.72 nm, respectively. Low magnification montages (LMM) and high magnification montages (HMM) were collected as standard procedure in SerialEM [31] via the ‘navigator’ GUI. Initially, LMM grid scans were recorded at a nominal magnification of 480× in EFTEM mode using the SerialEM ‘grid full montage’ option, with an overlay of 15%. Subsequently, HMM were recorded at magnifications of 5600× or 9300× with defocus ranges of −50 µm to −30 µm and an electron dose of 0.1 to 0.5 electrons/Å^2^ per individual image.

Tomographic tilt series were collected in three degree increments covering an angular range from −60° to +60° at a nominal magnification of 41,000× and defoci of −8 µm or −10 µm using SerialEM [31]. The total electron doses for the tilt series were kept between 90 and 110 electrons/Å².

#### 2.4.2 Image processing and visualisation

Tomographic reconstructions were calculated in IMOD [32] using weighted back-projection [33]. The visualisation of reconstructed tomograms was done in Amira 5.4 (Visage Imaging). For the final LMM and HMM collages shown (cf. Fig. 4), IMOD was used to improve the alignment of the images.

### 2.5 Accuracy of correlation calculation

#### 2.5.1 Correction of multi-channels fluorescent cryo microscopy images and determination of accuracy of alignment (σ^A^)

In order to correct the shift between the images of the different channels due to mechanical instabilities or chromatic shifts caused by the optics of the microscope, we used TetraSpeck microspheres, which are fluorescent in all three channels, as reference markers. To identify and determine the positions of the Tetraspeck fluorescent microspheres (fiducials) in all three channels with very high accuracies, we used algorithms which were originally developed for localisation microscopy (for details see Appendix A) [34,35]. An automated procedure for identifying objects that are fluorescent in all 3 channels was established and implemented in MATLAB (MathWorks). Tetraspeck microspheres were selected using a local search of fluorescent signal in the red, green and blue channels. Positions that did not show signal on all 3 channels within a radius of 500 nm were filtered out. This first criterion allowed the discrimination of the Tetraspeck from Ad5-488 viruses (only green fluorescence) or blue FluoSpheres (only blue fluorescence). A second criterion was set to exclude positions of Tetraspeck that are found to close together, *i.e*<500 nm. The remaining positions were used to determine and correct the shift between the different channels. The standard deviation gives a measure for the accuracy of the alignment *σ*^*A*^. In an additional filter step, outliers were removed by comparing the individual displacements with the mean shift.

For measurements where the shift was larger than 500 nm, a rough manual alignment was needed first. This was done by manually selecting one of the fiducials in all channels. The automated procedure used this information in the subsequent rough alignment.

Vitreous specimens behave as rigid bodies for the applied intensities. Therefore the shift was considered to be a global shift between the different channels. Image rotations are essentially non-existent in light microscopy as a single optical detection path is used. Local differences e.g. due to chromatic shifts were also neglected.

#### 2.5.2 Determination of accuracy of locating events of interest (σ^E^)

To determine the accuracy of locating an event of interest *σ*^*E*^ in the FM image, we fitted a two-dimensional Gaussian function with a linear background to the selected signals [34,35]. Using a Levenberg–Marquardt algorithm allowed an error estimation (standard error) of the position of each object, and thus directly provides the localisation accuracy.

#### 2.5.3 Fluorescent fiducial based coordinates transformation and determination of its accuracy (σ^C^)

The coordinate transformations based on fluorescent microsphere fiducials were assigned as described previously [24,25]. This procedure was performed using a MATLAB script based on software kindly provided by Martin Schorb (EMBL, Heidelberg, Germany). As inputs we used the two drift-corrected cryoLM images (blue and green channel), and the 2D projection EM images acquired at 5600× or 9300× nominal magnifications with calibrated pixel sizes of 3.65 nm and 2.10 nm (4.20 nm binned). Between 3 and 10 FluoSpheres were picked in each image. The precise coordinates of the signals of the fluorescent fiducials were determined using a 2D Gaussian fit, which was carried out on a fluorescence image after high-pass-filtering with a smooth cut-off of 70 pixels. The MATLAB control point selection tool was then used to determine the transformation between the FM and EM image based on the picked fiducials in both the FM and EM image.

For the calculation of the correlation accuracy *σ*^*C*^ was determined as described previously [24,25]. The position of one fiducial was left out for finding the transformation between the FM and the EM image. The fiducial position is then compared with the predicted position, and the displacement is measured. This is repeated for all fiducials selected in the images. The mean value of the displacements gives an estimate for the correlation accuracy *σ*^*C*^.

#### 2.5.4 Determination of total accuracy of correlation (σ^T^)

The overall uncertainty of the predicted position in the EM image is determined by all inaccuracies of the whole correlation procedure and the accuracy of locating the event of interest in the FM image. The inaccuracies *σ*^*A*^, *σ*^*E*^ and *σ*^*C*^ can be treated as independent errors. Thus, the overall uncertainty is given by *σ*^*T*^=√((*σ*^*A*^)^2^+(*σ*^*C*^)^2^+(*σ*^*E*^)^2^).

## 3 Results

### 3.1 Sample preparation for cryoCLEM

Adenovirus particles were labelled with Alexa-488 fluorophores (Ad5-488, for details see Section 2) thus providing a biological probe of known structure with a photon emission signal in the green spectrum [36,37].

Single-colour blue fluorescent microspheres (FluoSpheres) of 100 nm diameter were applied to the holey carbon support film on finder EM grids. These FluoSpheres were subsequently used to precisely correlate LM and EM images at high magnification as previously described for resin embedded sections of yeast samples [24,25]. Being spherical, the centre of the FluoSpheres can be defined accurately in any given orientation. Furthermore, the FluoSpheres were (i) selected to be single colour (here blue emission) and in their emission distinct from that of the label(s) of the target proteins (here green emission), (ii) to have a small enough diameter to provide a sufficient accuracy of correlation (e.g. 100 nm) and, (iii) to have a diameter large enough to be easily recognisable in EM amongst the sample environment and possible contaminants. Care was taken to deposit the FluoSpheres evenly and with high density across the grid. For this, the optimum was found to be 5–15 FluoSpheres in the high-magnification EM images of the region of interest.

Mammalian cells were grown on EM finder grids with an ideal cell density of one to two cells per grid square. Then, fluorescently labelled adenovirus particles were added to the cells and allowed to enter the cell. Next, the EM grids with adenovirus infected cells were vitrified by rapid plunge-freezing.

When imaging under cryo conditions, thermal drift of the fluorescence microscope stage presented a challenge. Since the blue channel (emission of the FluoSpheres) is ultimately used to correlate FM and EM images, and the relative location of the Ad5-488 viruses is deduced from that information, the overall correlation precision was severely limited. We therefore decided to add an internal fluorescence marker to monitor and correct the shifts between the fluorescence channels. To accomplish this, TetraSpeck fluorescent microspheres of 200 nm diameter were added to the growth medium of the cells on each grid just prior to plunge-freezing, *i.e*. in a similar manner to the gold fiducial markers routinely used for cryoET tilt series image alignment.

**Fig. 1:**
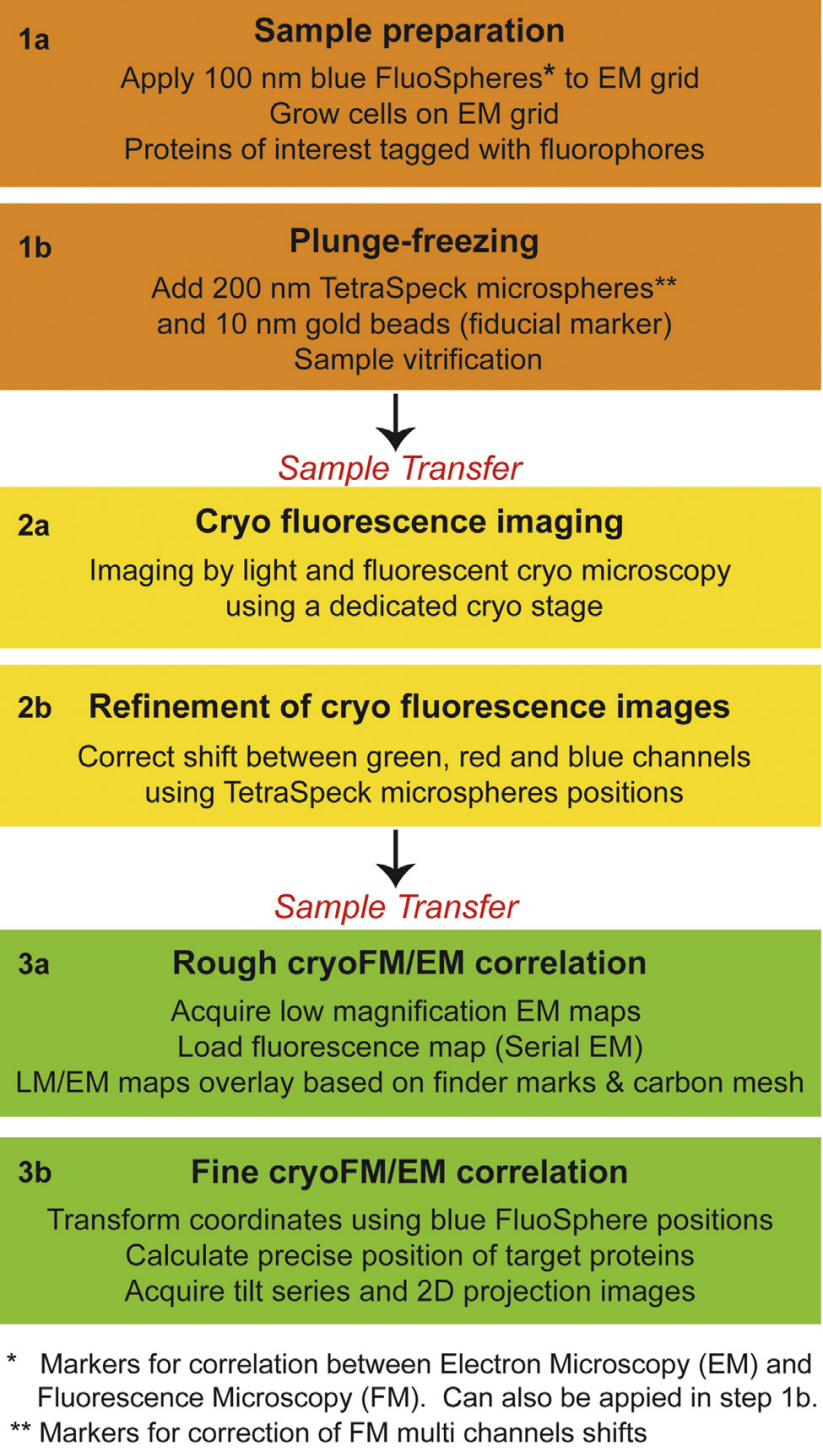
The major steps of correlated cryo light and electron microscopy using two independent correlation markers. The process begins with sample preparation (step 1a) and vitrification by rapid plunge-freezing (step 1b). The grids are subsequently imaged by light and fluorescence cryo microscopy (cryoFM) (step 2a) in different channels. Each set of FM images is merged according to the accurate positions of TetraSpeck microspheres (step 2b). The correlation with cryoEM data starts with a rough alignment (step 3a). Then, blue FluoSpheres are used as reference points to assign new coordinates to the EM images (step 3b). Finally, the location of the feature of interest is calculated and tomograms are acquired.

The diameter of the TetraSpeck microspheres was chosen to facilitate clear discrimination from the fluorescence intensity of the Ad5-488 virus. The abundance of TetraSpeck microspheres was optimised to avoid masking fluorescence from the virus particles. Additionally, the TetraSpeck diameter was selected to be clearly distinct from the 100 nm blue FluoSpheres to aid an unequivocal discrimination of the microsphere types during subsequent EM analyses. A density of 5 to 15 TetraSpeck microspheres of 200 nm diameter per grid square fulfilled the above requirements. Altogether, we devised an optimised workflow (Fig. 1) in order to overcome the current precision accuracy limits in correlative cryoFM/EM.

### 3.2 CryoLM data acquisition

Cryo light microscopy imaging was performed using the cryostage^2^, a second generation cryoLM sample stage designed in the group of W. Baumeister in Martinsried [18]. This stage was fitted onto the motorised x/y stage of an inverted light microscope, and imaging was performed using a 63× long working distance (LWD) air objective with a NA of 0.75.

**Fig. 2:**
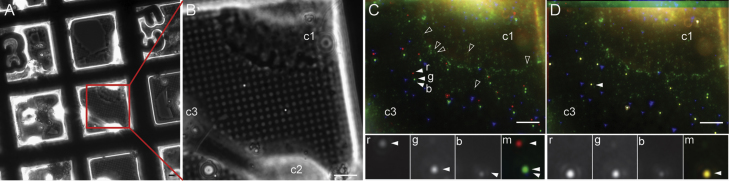
Cryo light and fluorescence imaging using TetraSpeck microspheres to monitor shifts between fluorescent channels. (A) Cryo phase contrast image of vitrified cells grown on EM grids acquired at low magnification (20×) and (B) at high magnification using a long working distance air objective (63×, NA 0.75). Three cells are present on the selected grid square (c1, c2, and c3). (C) Merge of cryo fluorescent images (FM) of the same area as in (B). Filled arrowheads point to the fluorescent signal of one TetraSpeck microsphere in red (r), green (g) and blue (b). Open arrowheads denote selected Ad5-488 virus fluorescence (green channel). The fluorescence signal of the TetraSpeck microsphere shows that the FM images are not aligned correctly due to stage drift (lower inset): 3098 nm and 787 nm shift in *X* and *Y* between red and blue signals; 449 nm and 148 nm shift in *X* and *Y* between green and blue signals. (D) Merge image of the cryoFM images after alignment according to the TetraSpeck microsphere positions. The fluorescent signal of the TetraSpeck microspheres appears yellow after correction indicating an accurate alignment. Correction was performed with 12.5 and 13.5 nm alignment precision between red/blue and green/blue images. Scale bars: 10 μm. (For interpretation of the references to color in this figure legend, the reader is referred to the web version of this article).

After plunge freezing, the grids were mounted under cryo conditions into dedicated FEI autogrid holder rims, in order to enhance the stability of the fragile grids to facilitate easier handling and transfer between the light and electron microscopes. As a first step, images in transmitted light mode, here phase contrast, were recorded using a low magnification lens (20×, NA of 0.4) in order to obtain a large field of view that includes finder marks on the grid (Fig. 2A). As a second step, grid squares with thin enough areas of cells, and thus more likely to be suitable for cryoET, were selected. An illustration is given in Fig. 2B, with cell 1 to be preferred over cell 2. Then, fluorescence images (Fig. 2C) in the red, green and blue channels were taken with the 63× LWD objective.

Using this imaging sequence, multiple examples were examined of which a more extreme test case is shown for illustration in Fig. 2. According to the fluorescence intensity, the location of individual Ad5-488 particles was easily identified, as well as the location of the TetraSpeck and blue FluoSpheres. As expected, the fluorescence of virus particles is weaker than that of the TetraSpeck microspheres (Fig. 2C and D), while the blue emission of the TetraSpeck microspheres was significantly weaker than that of the smaller blue FluoSpheres. Using algorithms developed for locating point-like fluorescent signals, it was possible to identify and determine the position of each TetraSpeck microsphere in the different channels (Fig. 2D, inset). The results are presented in Table 1. In the example shown in Fig. 2, the red image is shifted by 3098 nm and 787 nm in *X* and *Y*, respectively, as compared to the blue channel. Compared to other measurements, these shifts were among the largest observed values (Table 1). In most of the cases, the shift between the images for the cryoFM channels remained below 400 nm, as is the case for the example shown in Fig. 2D inset for green and blue channels.

Images were acquired manually in the order red, green and blue, in order to allow the focus to be optimised before each image acquisition. As a consequence, the drift between green and blue images is lower than between red and blue images.

Altogether, the measurements indicate that the cause of drift is complex, multifactorial and therefore hard to predict. In cryoLM each image set for the different fluorescent channels recorded should be aligned to each other, in order to yield the most accurate position before the translation of coordinates to cryoEM.

### 3.3 Multi channel cryoFM image alignment

We implemented a procedure for fluorescent channel alignment in MATLAB. All steps were fully or half automated for an efficient workflow, and accessible via a graphical user interface.

The initial step of the alignment and correlation procedure was to load the fluorescent images of the different channels into MATLAB. In most cases, it was convenient for further handling to crop the field of view to the region of interest (e.g. the part of a cell that is flat enough for EM). In those cases, care should be taken to include a sufficient number of alignment markers of each kind, i.e. TetraSpeck microspheres and blue FluoSpheres (see above).

**Fig. 3:**
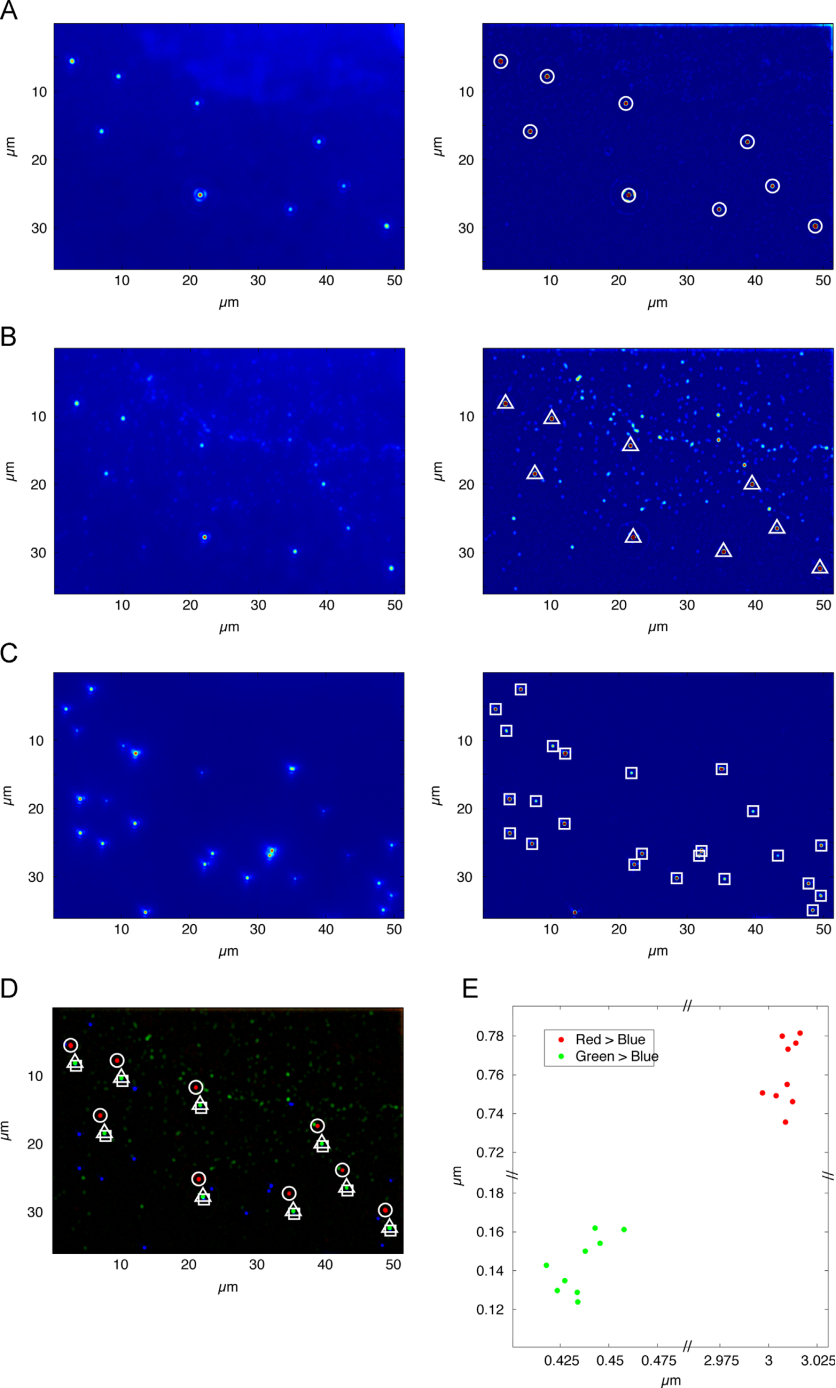
Detailed procedure for determining TetraSpeck microsphere positions in three fluorescence channels. Picking of microspheres in red (A) green (B) and blue (C) channels using an algorithm based on the *CLEAN* algorithm (Appendix A) and 2D Gaussian fit for precise position determination (left images, raw data; right images, processed data overlayed with found microspheres). Symbols in (C) indicate both FluoSpheres and TetraSpecks. (D) Filtered positions (symbols) for microspheres in all three channels. The circles, triangles and squares represent the selected Tetraspeck microspheres respectively in red, green and blue channels. (E) Scatter plot of the TetraSpeck microsphere displacement between the different channels. (For interpretation of the references to color in this figure legend, the reader is referred to the web version of this article).

In a second step, the shift between the different channels due to thermal or mechanical instabilities of the cryo sample stage, but also those induced by optical imperfections of the light microscope (e.g. chromatic shifts), are determined and corrected (Fig. 3). The steps of identifying the Tetraspeck fiducials, determining and correcting the shift between the channels, are fully automated. If the shift is very large (larger than 500 nm), a rough manual alignment may be needed first. This is simply done by selecting one of the Tetraspeck fiducials in all different channels to facilitate the automated correction process.

Since objects studied here are sufficiently small (<200 nm) with respect to the FWHM of the PSF of the light microscope, they can be treated as point-like objects. An algorithm for finding point-like objects on top of a variable background is used for initially identifying the beads (Fig. 3). Via a threshold (see Appendix A), the sensitivity of the algorithm can be adapted. The precise positions of the fiducials are automatically determined by fitting a two-dimensional Gaussian function with a linear background to the selected signals [34,35] (Appendix A). The positions are used to determine and correct the shift between the different channels. The standard deviation gives a measure for the accuracy of the alignment *σ*^*A*^.

Using this method, cryoFM images could be greatly improved with a remaining minor error of alignment, ranging from 5 to 33 nm (Table 1). Even very large shift values, as the one shown in Fig. 2, could be corrected with a precision *σ*^*A*^ of 15 nm.

### 3.4 Using cryoFM as a guideline for EM acquisition

In this section of the workflow (Fig. 1 step 3a), the feature of interest previously identified in LM is traced through a series of EM images of increasing magnification towards a final high magnification 2D projection image (as described in most CLEM methods). Importantly, automated EM data acquisition over large areas was used in order to (i) limit the electron dose, (ii) provide confidence in the correlation, and (iii) screen several regions of interest rapidly.

**Fig. 4:**
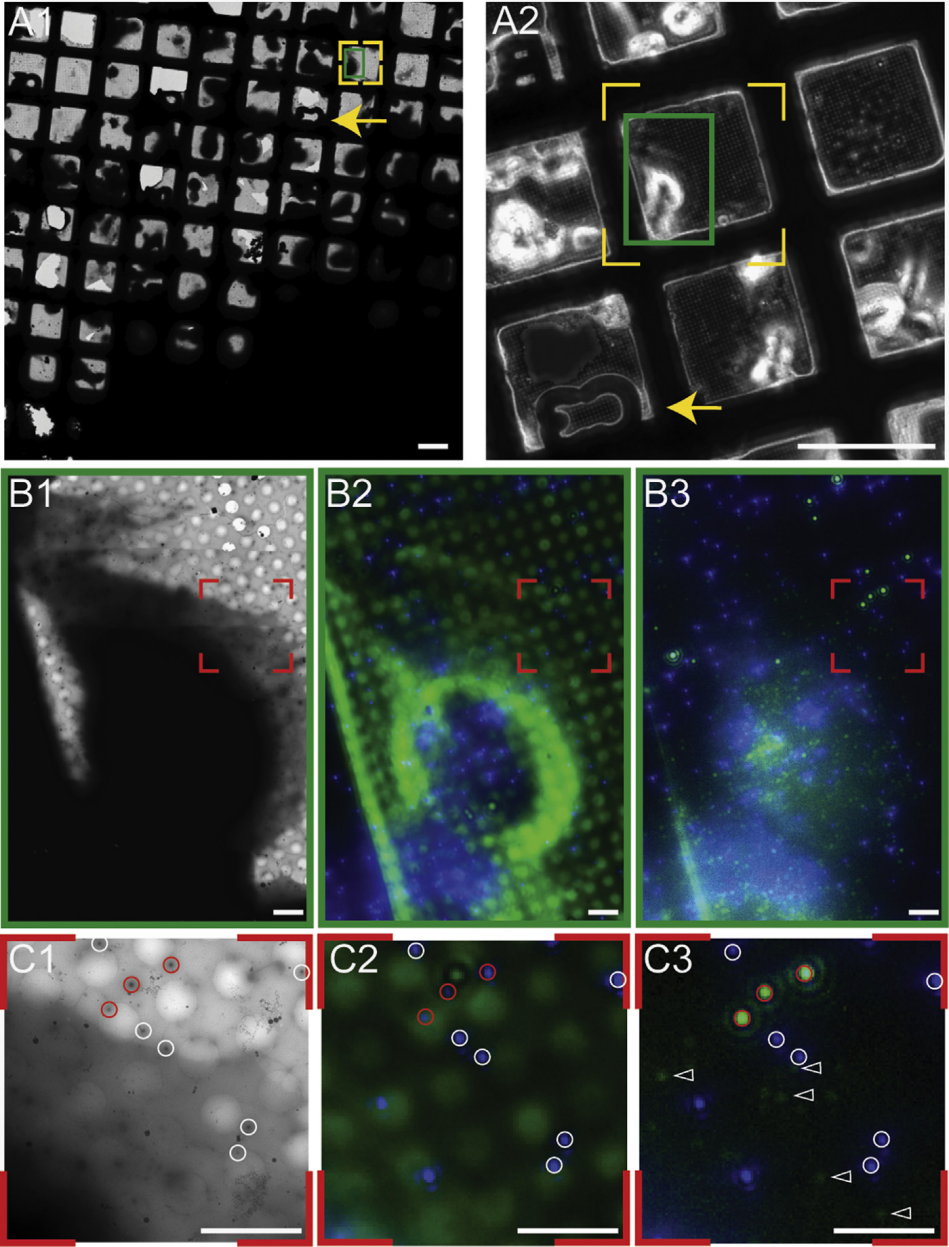
CryoLM and cryoEM correlation from low magnification to high magnification images of plunge-frozen U2OS cells infected with Ad5-488 labelled virus particles. (A1) A cryoEM low magnification montage (LMM) identifies the cell of interest (green rectangle in dashed yellow square). (A2) Shows a cryoLM phase contrast image for a subset of (A1). The arrows in (A1 and A2) point to the same grid marks. (B1) CryoEM high magnification montage (HMM) for the area indicated by the green rectangle in (A1/A2). (B2) Merged phase contrast (displayed in green) and blue FluoSpheres channel. (B3) Merged blue (FluoSpheres and TetraSpeck) and green (Ad5-488 virus and TetraSpeck) channel after multi-channel drift correction. The cell's shape, carbon mesh and FluoSpheres shown in B2 help in finding the relative orientation of HMM (B1) and cryoFM (B3) images. (C1-3) Zoom into areas indicated by red dashed squares in (B1-3), respectively. Highly fluorescent Ad5-488 viruses (marked by open arrowheads) were located in thin cellular areas. White and red circles indicate FluoSpheres and TetraSpeck microspheres respectively. Only microspheres close to the plasma membrane of the cell are circled in corresponding images. Scale Bars: (A1–A2) 100 μm; (B and C) 5 μm. (For interpretation of the references to color in this figure legend, the reader is referred to the web version of this article).

As part of the optimised workflow, the autogrid-mounted EM grids were transferred to our ‘Polara’ electron microscope in dedicated sample cartridges that allow the fitting of autogrid-mounted EM grids [18]. In order to re-locate previously selected grid squares, the entire grid was analysed using low magnification montage (LMM) imaging of the EM grid as available in SerialEM [31]. The cell of interest is located by the alphanumeric identifier marks of the finder grid (Fig. 4, arrow in A1 and A2).

Next, a third or half of the cell is imaged via a higher magnification montage (HMM) in the same fashion as the LMM (Fig. 4, B1). HMM maps typically covered an area of 80 µm×80 µm. Due to the beam rotations and alignment errors inherent when switching between low and higher magnification ranges in the EM, careful attention must be paid at this step. Significant efforts to optimise the EM column alignments were undertaken to ensure that the correlation between the LMM, HMM and the cryoFM images can be performed efficiently and reproducibly. At this level, the correlation is solely based on the recognition of large-scale visual features. Phase contrast cryoLM images provide useful information to enable the correct orientation of the images (Fig. 4: B1, B2, and B3). For instance, in cellular cryoEM unique features of the sample were often used for this level of correlation, such as the location of major cell protrusions or the plasma membrane position relative to the regular pattern of carbon holes in the support film. Typically, large areas of the cell were recorded to provide a sufficient number of landmarks.

As a consequence, by using a HMM, it is relatively straightforward to mark and target the thin area of cells which include features of interest and FluoSphere fine correlation markers. Thicker areas of the cells (thickness>~ 0.8 µm), like the region around the nucleus, which are inaccessible for cryoET as they cannot be penetrated by the electrons and therefore appear as black regions in the EM projection images (Fig. 4: B1), are easily detected and excluded already at the level of phase contrast LM images (Fig. 4: B2). This approach facilitated the identification of several highly fluorescent Ad5-488 viruses proximal to thinner plasma membrane edges; areas which were highly suitable for cellular cryoET. Additionally, the low sample thickness facilitates the identification of blue FluoSpheres as individual fluorescent signals. The images shown in Fig. 4: C1, C2, and C3 show a higher magnification of the same area visualised by a HMM, phase contrast cryoLM and FM, respectively.

Once the high magnification EM images of respective ROIs are recorded, the aim is to assign new coordinates between cryoEM and cryoLM in order to calculate more accurately the position of the particle of interest, here an Ad5-488 particle, and subsequently to record cryoET tilt series at the assigned location.

### 3.5 Post cryoEM acquisition fiducial based correlation

To correlate between the cryoLM and EM images, we used a previously described procedure implemented in MATLAB [24,25]. Based on the Control Point Selection Tool of MATLAB, the same FluoSpheres are selected in both images. These are used for the linear conformal transformation between the two coordinate systems. Then, the precise positions of the events of interest identified in the fluorescence image can be plotted into the EM image.

To determine the error of the correlation precision, we used a modified version of the correlation procedure described previously [24,25], omitting the correction for the sample deformation between LM and EM required for plastic embedded specimens. Briefly, the 100 nm FluoSphere fiducials were used as reference points to assign new coordinates to EM images. For each calculation of correlation error, one fiducial is excluded from the calculation of the transform. This bead is treated as the object of interest and its position predicted within the EM projection image. The calculated position is then compared to the actual position in the EM image and the error of the prediction determined. Performing this operation for all beads in the image along with a statistical analysis it is possible to calculate an average error for the correction of both images. This method is illustrated here using two different samples (Table 2), the first being an EM grid of vitrified FluoSpheres and TetraSpeck microspheres only. For the second sample, plunge-frozen cells infected with Ad5-488 labelled viruses, prepared as described before, were examined. Additionally, images were acquired at two different magnifications in the electron microscope and the procedure repeated. It should be noted that upon changing to lower EM magnifications, more blue FluoSpheres are imaged in the larger field of view thus providing a greater number of measurements and more accurate statistics. The calculated correlation errors (Table 2) ranged between a minimum of 12.5 nm and a maximum of 42.5 nm. This is in the range of the values published previously for plastic-embedded specimens [24,25].

### 3.6 Calculation of overall correlation precision

The overall uncertainty of the predicted position in the EM image was determined by the inaccuracy of the alignment of the different FM channels *σ*^*A*^, the precision of determining the position of the event of interest *σ*^*E*^ and the accuracy of the correlation of the fluorescence *σ*^*C*^. The latter, *σ*^*A*^, *σ*^*E*^ and *σ*^*C*^, can be treated as independent errors. Thus, the overall uncertainty is given by *σ*^*T*^=√((*σ*^*A*^)^2^+(*σ*^*C*^)^2^+(*σ*^*E*^)^2^).

The alignment inaccuracy of the different FM channels *σ*^*A*^ and *σ*^*C*^ was described in Sections 3.2 and 3.5, respectively. *σ*^*E*^ is described below in Section 3.7.

### 3.7 Application to fluorescently labelled adenovirus particles

**Fig. 5:**
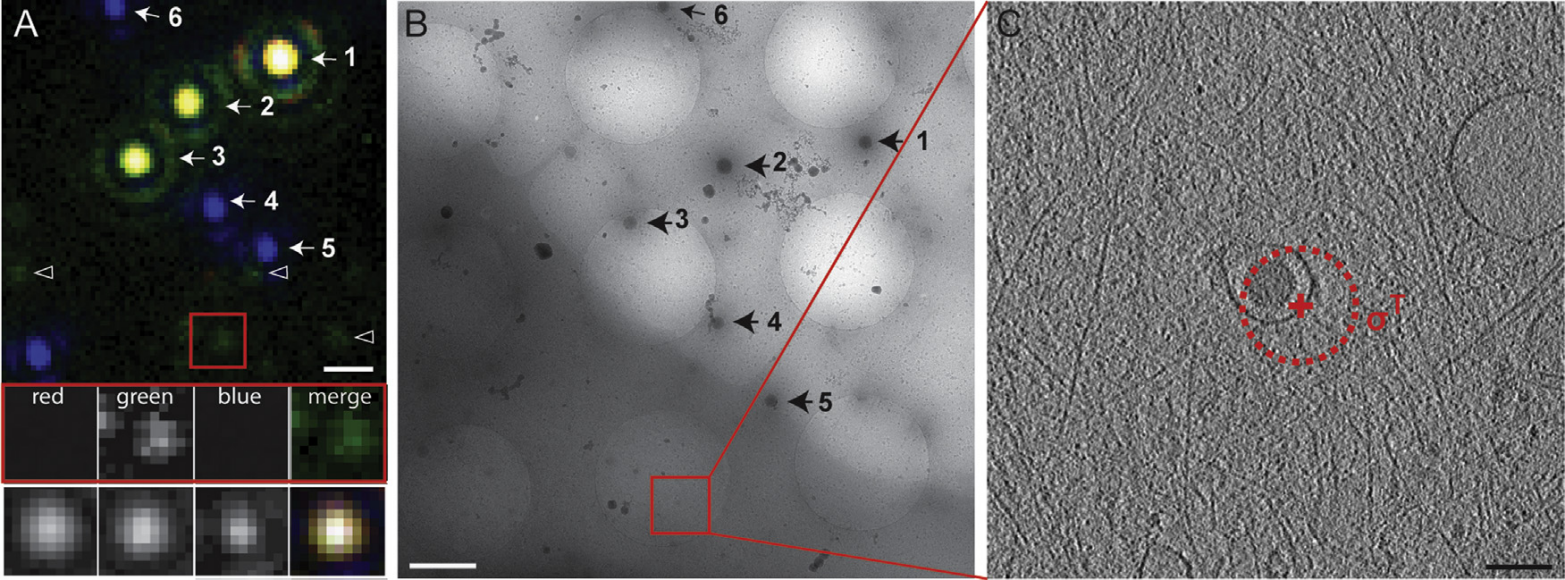
High precision correlation applied to the location of adenovirus particles in vitrified cells. (A) Fluorescent cryo microscopy image of plunge-frozen U2OS cells infected with green fluorescent Ad5-488 viruses. Merged fluorescent image after channel alignment using TetraSpeck microsphere fluorescence. Upper row of insets shows magnified images of the RGB channels and merge for the Ad5-488 virus marked by the red square. The lower row of insets shows magnified images of TetraSpeck number 3 illustrating the alignment accuracy (*σ*^*A*^=11.8 nm) across the three FM channels. The Ad5-488 signal marked by the red square (*σ*^*E*^=75 nm) in the region of interest (ROI) was targeted for cryoET. (B) Medium magnification cryoEM projection image of the ROI shown in (A). The correlation between (A) and (B) was performed using the FluoSpheres numbered 4, 5 and 6 (*σ*^*C*^=43.9 nm) as reference points. The subarea selected for tomography is indicated by the red square. (C) Computational slice through tomogram for the subarea indicated in (B) shows an adenovirus particle in an endosome, surrounded by actin filaments. The overall correlation error (*σ*^*T*^=87.5 nm) is plotted as radius of the red dashed circle centred on the calculated coordinates of the targeted Ad5-488 signal. Scale Bars: (A, B) 1 μm; (C) 100 nm. (For interpretation of the references to color in this figure legend, the reader is referred to the web version of this article).

In order to verify that the devised workflow can be successfully applied with the same high accuracy to identify a biological object of interest labelled with a different fluorescent colour than that of the FluoSpheres, we tested it for locating Ad5-488 virus particles inside cellular specimens. As the viral particles emit in a different channel than FluoSpheres, the error calculation involves an additional step. For each event of interest, the inaccuracy of the alignment of the different channels (*σ*^*A*^, see Sections 3.1 and 3.2) and the inaccuracy of the fiducial based correlation were determined (*σ*^*C*^, see Section 3.5). Additionally, we determined the localisation inaccuracy of the Ad5-488 spot (*σ*^*E*^) being limited by the width of the PSF and the number of photons detected (see Section 2). The results are summarised in Table 3. For three examples, virus particles were found with an overall correlation error *σ*^*T*^ of 51.1 nm, 73.0 nm and 87.9 nm. Fig. 5 illustrates test case number 2, for which the overall calculated correlation error, i.e. 87.9 nm, was displayed on top of the cryoEM image. In this particular case, the same region of interest as described in Fig. 4 was used to correlate cryoFM and cryoEM images. Several fluorescent Ad5-488 particles were located close to blue FluoSpheres in a thin area of the cell. A virus with relatively high green fluorescence in this ROI was targeted (red square in Fig. 5A), and three FluoSpheres were used (marked in Fig. 5 as 4, 5 and 6) as reference points to assign new coordinates to the EM image. The location of the virus particle was then calculated on the high magnification EM image and the location reported to the tomogram slice shown in Fig. 5C. Tomography helped to unveil the structure and identity of the targeted feature, which at the level of the EM projection image had remained ambiguous due to the overlay of the virus with numerous cellular structures. The tomogram slice shown in Fig. 5C clearly reveals an adenovirus particle located in an endosomal vesicle, surrounded by the cellular actin network.

## 4 Discussion

To explore the full potential of cellular cryoET, we investigated the correlation accuracy that can be achieved routinely between cryoFM and cryoEM of vitreous whole cells. Recording and correlating images of plunge-frozen samples is a challenge and a field of active development. On the LM side, cryo sample stages are less stable than imaging stages dedicated for room temperature, and the choice of objectives or other system elements is more restricted for cryo imaging [8]. On the EM side, the inaccuracy of column alignments, beam and image shift calibrations can be substantial, thereby inducing shifts in *X*, *Y* between magnifications and limiting the correlation precision and efficiency. Correlation software that can assist the process is under development, but the accuracy of correlation has remained limited to micrometre accuracy [9,28]. Despite these challenges, the technique of cryoCLEM offers a unique opportunity to gain functional insights into subcellular processes by combining the virtues of both cryoLM and cryoEM. When applied to whole, vitrified cells, the process of interest can be studied in its most native context, and the structural integrity is preserved over a large resolution range.

Here, we designed and implemented a cryoCLEM pipeline that enabled a more accurate correlation between cryoLM and cryoEM images by introducing a number of dedicated correlation markers for different levels of alignment. Additionally, the contribution of each step in the workflow to the overall correlation accuracy was determined. To achieve this, we combined tools that are already known but have never been put together to perform cryoCLEM. The use of fluorescent microspheres (FluoSpheres) had been put forward for resin embedded sections [24,25]. Also TetraSpeck microspheres are commonly used in fluorescence microscopy in order to align fluorescence channels accurately and to correct for chromatic misalignments. A further important tool was the precise determination of positions of point-like objects in FM taken from localisation microscopy [12–14,35]. By using the location of TetraSpeck microspheres of 200 nm diameter, the cryoFM images could be greatly improved for cryoCLEM applications. Image shifts ranging from a few to several thousand nanometres between different images were correctable with a precision *σ*^*A*^ in the 20 nm range. The choice of TetraSpeck was critical in the discrimination between microspheres and target fluorescent molecules, such as Ad5-488 viruses. Measurements over dozens of data sets also clearly demonstrated that, as expected, due to the cryo stage instabilities, the drift varies significantly between different sets of images independent of the thickness of ice or the type of samples. Therefore, each cryoFM image set for the different fluorescent channels should be corrected relative to each other in order to yield the most accurate position before translating the coordinates to cryoEM.

Automated EM data acquisition then allowed us to re-locate the ROI identified in cryoFM images. To accomplish this, 2D projection image montages in SerialEM of increasing magnification were used. Throughout our experiments, we found that phase contrast cryo images yielded a number of benefits. As described previously [19], these images allowed for easy identification of the ROI on the EM grid according to the grid finder marks and the assessment of the ice thickness, and therefore a rapid screening of areas suitable for cryoEM data acquisition. By adapting for cryo conditions the fiducial based correlation method described previously for resin embedded samples [24,25], we could assign coordinates between LM and EM based on blue FluoSpheres and record tomograms of structures identified by FM.

In this study, Adenoviruses were used as model macromolecular complexes to monitor the resulting accuracy of the correlation. This object has the advantage that its structure has been characterised previously [36,37], and that the particles have a diameter of 90 nm, i.e. significantly below the FWHM of the PSF of the used optical microscope setup.

Application of our method to Adenovirus capsids clearly shows that nanometre scale correlation is attainable with frozen hydrated specimens using our workflow. Positions of Ad5-488 on the EM grid could be determined with an overall correlation accuracy in the range of ~60 nm. This is an improvement of almost one order of magnitude compared to the conventional localisation precision of ~450 nm for our cryoLM setup. Determination of the localisation precision of each object additionally allows an estimation of the overall correlation accuracy *σ*^*T*^ for each object individually. It should be noted that the precise localisation of fluorescent objects is only applicable for those that have a size that is substantially smaller than the FWHM of the PSF of the optical imaging system. Only then they can be treated as point-like objects, which is a necessary condition for the precise position determination. Larger structures would require a more complex model function that also includes information about their shape instead of a fit with a 2D Gaussian.

To address the limitation of the localisation accuracy of the event of interest *σ*^*E*^, one could use a more sensitive camera for FM acquisitions to increase the number of detected photons and decrease the integration time. Purely increasing the integration time might lead to significantly blurred signals due to the instability of the cryoLM imaging system. In our case, the integration time for one image was 2–3 s, which in some cases caused a mean anisotropy of the PSF of 0.06 (see Supplementary Fig. S1). An additional prospect would be to use immersion lenses with a higher NA in cryo conditions, but although such experimental setups have been reported [38], these lenses are not commercially available yet.

The inaccuracies linked to the FM image alignments *σ*^*A*^ (avg. of 15 nm) or the fiducial based coordinate transformation *σ*^*C*^ (avg. of 38 nm) are minor errors compared to the localisation accuracy *σ*^*E*^ for the Ad5-488 particles (avg. of 63 nm). As an example, we calculated the overall correlation precision for a fluorescent microsphere of 100 nm diameter (from data set case 3 in Table 3), i.e. similar to the size of a typical virus particle. In this case, since the fluorescence intensity is much higher, the location of the microsphere in cryoFM could be determined with an accuracy *σ*^*E*^ of ~5 nm (avg. for multiple measurements). With *σ*^*A*^ of ~7.8 nm and *σ*^*C*^ of ~12.2 nm the overall correlation precision *σ*^*T*^ is ~15 nm. This illustrates that the overall correlation accuracy *σ*^*T*^ is mainly limited by the number of detected photons for the event of interest in cryoFM. The technique presented here has the capability of reaching molecular precision for locating objects in cryoEM images identified with cryoFM.

## 5 Conclusions

In summary, by using a novel workflow for cryoCLEM, we were able to identify plunge frozen fluorescently labelled viruses in mammalian cells to nanometre scale precision. This method opens new perspectives, such as studying structurally uncharacterized macromolecular complexes in their native form and cellular context. The method will be particularly useful to identify rare co-localisation events in the cell. Moreover, the method can be implemented in various CLEM implementations, with the use of different fluorophores or labelling strategies in order to improve the localisation precision.

## Figures and Tables

**Table 1:** Measurement and correction of shifts for multi fluorescence cryo microscopy imaging.

*Merged images*	*Axis*	Measured shifts [nm]			Alignment precision *σ*^*A*^ [nm]		
		*Max*	*Min*	***Average***	*Max*	*Min*	***Average***
Green/Blue	*X*	1417	3	**142**	29	8	**17**
	*Y*	357	4	**156**	30	13	**20**

Red/Blue	*X*	3098	8	**415**	25	5	**13**
	*Y*	868	1	**309**	33	9	**15**

For each set of images, the shift between red/blue and green/blue fluorescence images was measured and corrected according to TetraSpeck microspheres of 200 nm diameter. The alignment precision *σ*^*A*^ is given in nm. Data corresponding to the maximum, minimum and average values were measured on 8 data sets recorded on plunge-frozen cells infected with Ad5-488 viruses and 8 data sets recorded on a plunge-frozen microsphere sample. A total of 3 to 20 TetraSpeck microspheres were used per image for the calculations.

**Table 2:** CryoCLEM correlation precision using FluoSpheres as correlation markers.

Data sets	CryoEM *nominal mag.*	Fiducial based correlation	
		*No. of fiducials*	***Precision σ***^***C***^ [nm]
Sample 1	9300	9	**32.5**
	5600	10	**45.5**

Sample 2	9300	5	**12.2**
	5600	4	**30.3**

Measurements were performed on four data sets acquired on a plunge-frozen EM-grid with only FluoSpheres and TetraSpeck microspheres (sample 1). Sample 2 additionally contained plunge-frozen cells infected with Ad5-488 viruses. 4 to 10 FluoSpheres of 100 nm were picked manually on each image and used as fiducials for coordinate transformations. The correlation precision *σ*^*C*^ is calculated as described by Kukulski et al. [23] (see Section 2).

**Table 3:** CryoCLEM correlation precision for Ad5-488 virus particles.

Data sets	CryoFM, alignment *σ*^*A*^			Fiducial correlation *σ*^*C*^	Event of interest *σ*^*E*^	**Total*****σ***^***T***^ [nm]
	*X*	*Y*				
Ad5-488 case 1	7.4	8.1	(3)*	12.2 (5)**	49	**51.1**
Ad5-488 case 2	8.4	15.1	(7)*	43.9 (3)**	75	**87.9**
Ad5-488 case 3	17.5	35.4	(5)*	19.4 (5)**	65	**73**

Three cryoLM and cryoEM data sets were acquired on a vitrified cellular sample infected with Ad5-488 viruses. TetraSpeck microspheres of 200 nm diameter were used to measure the alignment precision *σ*^*A*^ between green and blue images. FluoSpheres of 100 nm were used for the fiducial based alignment. The correlation inaccuracy *σ*^*C*^ was calculated and the inaccuracy of locating the event of interest *σ*^*E*^ was measured by applying a 2D Gaussian fit. The overall total correlation inaccuracy for each event of interest was calculated as *σ*^*T*^=√((*σ*^*A*^)^2^+(*σ*^*C*^)^2^+(*σ*^*E*^)^2^). The number of TetraSpeck* microspheres and FluoSpheres** used for each experiments are indicated in brackets.

## Appendix A Algorithm for identifying point-like objects

The principle of the algorithm that we used for finding objects is similar to the CLEAN algorithm used in radio astronomy [S1, S2]. The image is first bandpass filtered to suppress noise as well as larger features in the background; thus, with appropriate filter settings only point-like objects remain. In a second step maxima (brightest objects) in the image are identified via a threshold and their positions noted down. The threshold image is convolved with a Gaussian with a FWHM similar to the PSF of the microscope and subtracted from the filtered image. In the resulting image the bright objects are removed revealing objects with lower intensity in their vicinity, which can then be identified in the following step at a lower threshold. This procedure is repeated until the value of the maximum drops below a pre-set threshold, (cf. Section 3.3). The FWHM of the PSF can be determined using fluorescent microspheres as mentioned in Section 2.3. This is critical for the above step to avoid artefacts during the *CLEAN* procedure (e.g. rings around the position of the removed object if FWHM of applied Gaussian too small).

[S1] B. Clark, An efficient implementation of the algorithm ‘CLEAN’, Astron. Astrophys., 89 (1980) 377–378.

[S2] J. Högbom, Aperture synthesis with a non-regular distribution of interferometer baselines, Astron. Astrophys. Suppl. S., 15 (1974) 417–426.
