# Supplementary figures and images for: High-precision correlative fluorescence and electron cryo microscopy using two independent alignment markers
Source: Ultramicroscopy. 2014 Aug;143(100):41–51. doi: 10.1016/j.ultramic.2013.10.011 (PMC4045203; doi:10.1016/j.ultramic.2013.10.011)

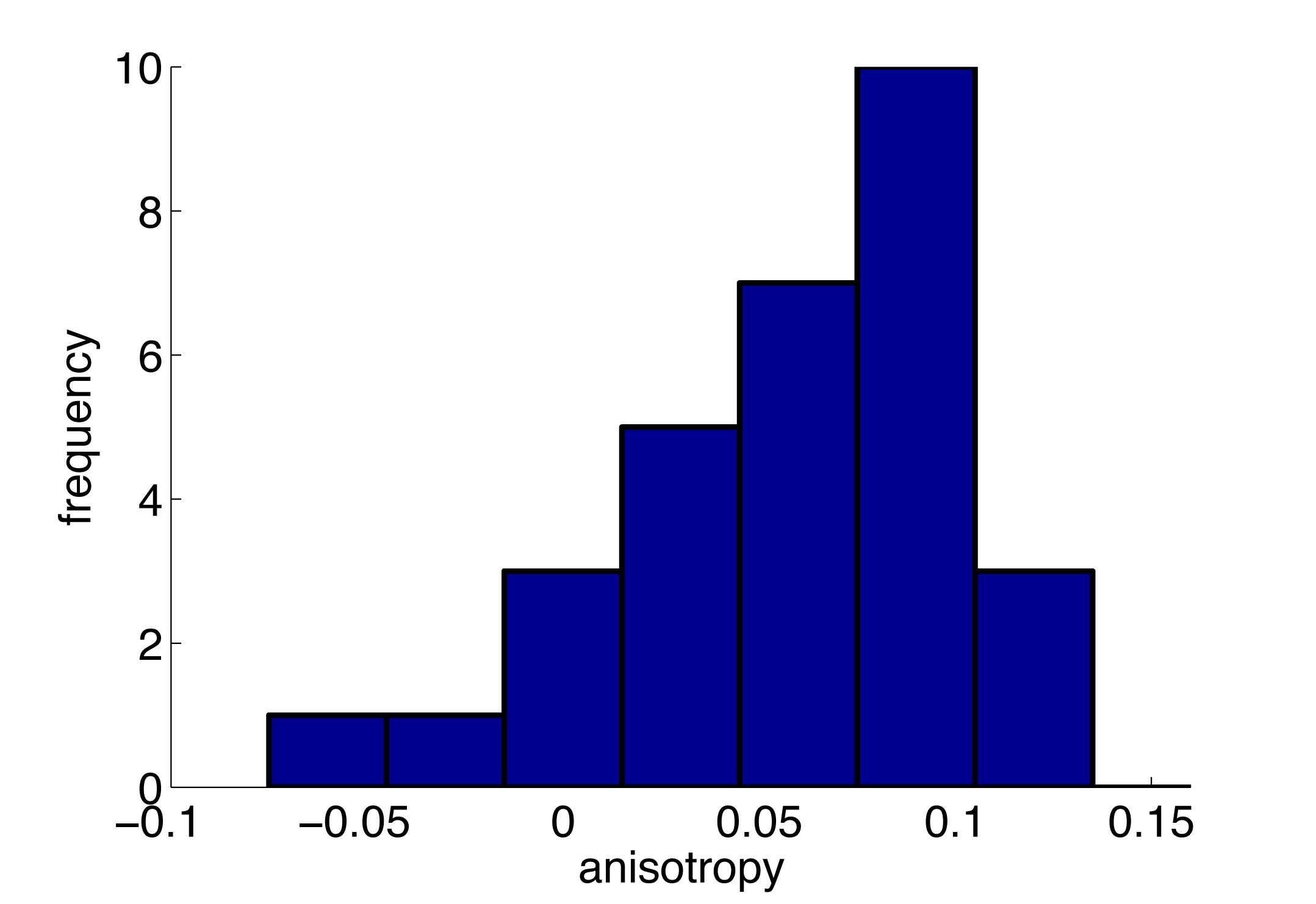

Supplement: Supplementary file 1 — Supplementary Data [file mmc1.zip › suppl_FigS1.tif]
